# Heart Disease and Cancer Deaths — Trends and Projections in the United States, 1969–2020

**DOI:** 10.5888/pcd13.160211

**Published:** 2016-11-17

**Authors:** Hannah K. Weir, Robert N. Anderson, Sallyann M. Coleman King, Ashwini Soman, Trevor D. Thompson, Yuling Hong, Bjorn Moller, Steven Leadbetter

**Affiliations:** Author Affiliations: Robert N. Anderson, Division of Vital Statistics, National Center for Health Statistics, Centers for Disease Control and Prevention, Hyattsville, Maryland; Sallyann M. Coleman King, Yuling Hong, Division for Heart Disease and Stroke Prevention, National Center for Chronic Disease Prevention and Health Promotion, Centers for Disease Control and Prevention, Atlanta, Georgia; Ashwini Soman, Northrop Grumman Corporation, Atlanta, Georgia; Trevor D. Thompson, Steven Leadbetter, Division of Cancer Prevention and Control, National Center for Chronic Disease Prevention and Health Promotion, Centers for Disease Control and Prevention, Atlanta, Georgia; Bjorn Moller, Department of Registration, Cancer Registry of Norway, Oslo, Norway.

## Abstract

**Introduction:**

Heart disease and cancer are the first and second leading causes of death in the United States. Age-standardized death rates (risk) have declined since the 1960s for heart disease and for cancer since the 1990s, whereas the overall number of heart disease deaths declined and cancer deaths increased. We analyzed mortality data to evaluate and project the effect of risk reduction, population growth, and aging on the number of heart disease and cancer deaths to the year 2020.

**Methods:**

We used mortality data, population estimates, and population projections to estimate and predict heart disease and cancer deaths from 1969 through 2020 and to apportion changes in deaths resulting from population risk, growth, and aging.

**Results:**

We predicted that from 1969 through 2020, the number of heart disease deaths would decrease 21.3% among men (–73.9% risk, 17.9% growth, 34.7% aging) and 13.4% among women (–73.3% risk, 17.1% growth, 42.8% aging) while the number of cancer deaths would increase 91.1% among men (–33.5% risk, 45.6% growth, 79.0% aging) and 101.1% among women (–23.8% risk, 48.8% growth, 76.0% aging). We predicted that cancer would become the leading cause of death around 2016, although sex-specific crossover years varied.

**Conclusion:**

Risk of death declined more steeply for heart disease than cancer, offset the increase in heart disease deaths, and partially offset the increase in cancer deaths resulting from demographic changes over the past 4 decades. If current trends continue, cancer will become the leading cause of death by 2020.

**MEDSCAPE CME**

Medscape, LLC is pleased to provide online continuing medical education (CME) for this journal article, allowing clinicians the opportunity to earn CME credit.

This activity has been planned and implemented through the joint providership of Medscape, LLC and *Preventing Chronic Disease*. Medscape, LLC is accredited by the American Nurses Credentialing Center (ANCC), the Accreditation Council for Pharmacy Education (ACPE), and the Accreditation Council for Continuing Medical Education (ACCME), to provide continuing education for the healthcare team.

Medscape, LLC designates this Journal-based CME activity for a maximum of 1.00 ***AMA PRA Category 1 Credit(s)™***. Physicians should claim only the credit commensurate with the extent of their participation in the activity.

All other clinicians completing this activity will be issued a certificate of participation. To participate in this journal CME activity: (1) review the learning objectives and author disclosures; (2) study the education content; (3) take the post-test with a 75% minimum passing score and complete the evaluation at http://www.medscape.org/journal/pcd; (4) view/print certificate.

Release date: November 17, 2016; Expiration date: November 17, 2017

**Learning Objectives**

Upon completion of this activity, participants will be able to:

1. Distinguish the overall effects of risk reduction and population growth and aging on the projected number of heart disease and cancer deaths to the year 2020, based on a study of mortality data, population estimates, and population projections
2. Identify factors contributing to the decline in heart disease risk
3. Identify factors associated with the increase in cancer risk

**EDITOR**

Rosemarie Perrin

Editor, Preventing Chronic Disease

Disclosure: Rosemarie Perrin has disclosed no relevant financial relationships.

**CME AUTHOR**

Laurie Barclay, MD

Freelance writer and reviewer, Medscape, LLC

Disclosure: Laurie Barclay, MD, has disclosed the following relevant financial relationships:

Owns stock, stock options, or bonds from: Pfizer

**AUTHORS**

Hannah K. Weir, PhD

Division of Cancer Prevention and Control, National Center for Chronic Disease Prevention and Health Promotion, Centers for Disease Control and Prevention, Atlanta, Georgia

Disclosure: Hannah K. Weir, PhD, has disclosed no relevant financial relationships.

Robert N. Anderson, PhD

Division of Vital Statistics, National Center for Health Statistics, Centers for Disease Control and Prevention, Hyattsville, Maryland

Disclosure: Robert N. Anderson, PhD, has disclosed no relevant financial relationships.

Sallyann M. Coleman King, MD, MSc

Division for Heart Disease and Stroke Prevention, National Center for Chronic Disease Prevention and Health Promotion, Centers for Disease Control and Prevention, Atlanta, Georgia

Disclosure: Sallyann M. Coleman King, MD, MSc, has disclosed no relevant financial relationships.

Ashwini Soman, MPH

Northrop Grumman Corporation, Atlanta, Georgia

Disclosure: Ashwini Soman, MPH, has disclosed no relevant financial relationships.

Trevor D. Thompson, BS

Division of Cancer Prevention and Control, National Center for Chronic Disease Prevention and Health Promotion, Centers for Disease Control and Prevention, Atlanta, Georgia

Disclosure: Trevor D. Thompson, BS, has disclosed no relevant financial relationships.

Yuling Hong, MD, PhD

Division for Heart Disease and Stroke Prevention, National Center for Chronic Disease Prevention and Health Promotion, Centers for Disease Control and Prevention, Atlanta, Georgia

Disclosure: Yuling Hong, MD, PhD, has disclosed no relevant financial relationships.

Bjorn Moller, PhD

Department of Registration, Cancer Registry of Norway, Oslo, Norway

Disclosure: Bjorn Moller, PhD, has disclosed no relevant financial relationships.

Steven Leadbetter, MS

Division of Cancer Prevention and Control, National Center for Chronic Disease Prevention and Health Promotion, Centers for Disease Control and Prevention, Atlanta, Georgia

Disclosure: Steven Leadbetter, MS, has disclosed no relevant financial relationships.

## Introduction

For most of the last century, the leading cause of death in the United States, as measured by actual deaths, was heart disease, followed by cancer (1). Cancer overtook heart disease to become the leading cause of death in 1 state (Alaska) in 1993, 2 states in 2000, 8 states in 2005, and 23 states in 2010, although the trend slowed or stopped in recent years (2,3).

The age-standardized death rate approximates the population’s risk of dying from a given cause and is used to compare risk of death between populations or within a population over time. Declining death rates indicate that the overall risk to the population of dying from heart disease or cancer decreased. However, age-standardized death rates do not convey the full extent of the burden of these diseases, because they effectively remove the influence of demographic changes related to population growth and changing age structure. Although the age-standardized death rate for heart disease began to decline in the late 1960s and for all cancers combined some 20 years later, the overall number of heart disease deaths declined and the number of cancer deaths increased (1,4).

The number of deaths is a function of the population risk of being diagnosed and dying from that cause and the size and age structure of the population. The risk of death from heart disease and cancer generally increases with age, and over the past several decades the US population increased, particularly in the age group 65 years or older (5). These demographic changes are forecast to continue into this century as the cohort born after World War II, with increased longevity than earlier cohorts, enters the age groups most at risk of dying from heart disease and cancer.

The objective of this study was to use mortality data, current population estimates, and population projections to predict age-standardized death rates and death counts for heart disease and cancer from 1969, around the peak of heart disease death rates (risk), through 2020 and to apportion changes in deaths resulting from population risk reduction, population growth, and population aging (ie, shift in age distribution toward older ages and increased longevity).

## Methods

### Source of data

We obtained mortality data from 1969 through 2014 from the National Vital Statistics System (6).The underlying cause of death was assigned according to the *International Classification of Disease* (ICD) in use at the time of death, converted to ICD-10 (*International Classification of Disease*, *Revision 10*), and recoded to ensure comparability over time (7). For these analyses, we defined heart disease as rheumatic heart disease (I00–I09), hypertensive heart disease (I11), hypertensive heart and kidney disease (I13), acute myocardial infarction (I21–I22), other ischemic or coronary heart disease (I20, I23–I25), atrial fibrillation (I48), other arrhythmias (I47, I49), heart failure (I50), and other heart disease (I26–I146, I51); we defined cancer as malignant neoplasms (ICD-10: C00–97).

Bridged single-race population estimates based on the 2010 US Census were available through the Surveillance, Epidemiology, and End Results (SEER) Program (8). We obtained data on population projections of the resident population by race, age, and sex from 2015 through 2020 from the US Census Bureau’s Population Projections Program (9). Population estimates and projections were used as the denominators in rate calculations.

### Trends in death rates, 1969–2014

We performed statistical analyses using SEER Stat software, version 8.3.2 (Surveillance Research Program [http://seer.cancer.gov/seerstat/]), calculating averaged, annual age-standardized death rates per 100,000 population and standardized to the 2000 population, by sex and race (all, white, black). We estimated trends in death rates from 1969 through 2014 using joinpoint regression (Joinpoint Trend Analysis Software, version 4.2.0.1, http://surveillance.cancer.gov/joinpoint/), where a maximum of 5 joined straight-line segments were fit on a logarithmic scale to the trends in annual death rates. We described the resulting trends by the slope of each line segment as the annual percentage change (APC), using *t* tests (2-sided, *P* < .05) to assess whether the APCs were significantly different from zero. We used the terms *increase* or *decrease* to describe significant trends and *stable* to describe nonsignificant trends.

Methods for projecting cancer death rates and counts are described in detail elsewhere (10). Briefly, to project age-standardized death rates and counts for 2015 through 2020, we used Nordpred software (11,12), which uses an age-period–cohort regression model with data aggregated into six 5-year calendar periods (1985–1989, 1990–1994, 1995–1999, 2000–2004, 2005–2009, 2010–2014) and 15 age groups (15–19, 20–24, 25–29, 30–34, 35–39, 40–44, 45–49, 50–54, 55–59, 60–64, 65–69, 70–74, 75–79, 80–84, ≥85 y). Separate models were constructed for heart disease causes of death and for leading cancer causes of death, by sex for all races combined. We based projections for all heart disease deaths and all cancer deaths on summed estimates among the individual disease categories. We obtained predicted death counts and age-standardized death rates by applying estimated age-specific death rates to the population projections for 2015 through 2020.

Methods to apportion the relative contribution to changes in the total number of new heart disease or cancer deaths each year that can be attributed to changes in population risk (including changes in diagnosis and treatment practices) and demographic changes related to population size and age structure are described elsewhere (10). Briefly, we generated 3 sets of data for each death year from 1969 through 2020. Baseline was defined as the number of deaths from heart disease or cancer that occurred in 1969 for men and women separately. We generated one set of data for the total number of cancer deaths that would have occurred each year if the population size and age structure remained the same as it was in 1969; this set reflects the effect of changes in population risk and is similar to the age-standardized death rate. A second set of data was generated for the total number of deaths that would have occurred if the age structure had remained the same as it was in 1969; this set reflects the effect of changes in risk and population growth. A third set of data was generated for the observed number of deaths that actually occurred and thus reflects the combined impact of changes in population risk, growth, and aging. The yearly difference between each set of death counts denotes the relative contribution to the overall change in the number of deaths since 1969 attributed to population risk, growth, and aging. A decline in risk results in negative death counts, because fewer deaths are attributed to risk compared with 1969, the baseline year.

## Results

The percentage change in death rates for heart disease declined among men (68.4%) and women (67.6%) (Table 1). By race and sex, the percentage decline was 68.8% among white men, 67.6% among white women, 59.4% among black men, and 63.8% among black women. The APCs for heart disease death rates for all races combined declined from 1969 through 2014 for men and women. By race and sex, the APC was stable in white men from 2010 through 2014 and from 2011 through 2014 for black men and women. The APC continued to decrease among white women through 2014.

**Table 1:** Age-Standardized Death Rates and Overall Percentage Change and the Annual Percentage Change in Age-Standardized Death Rates by Joinpoint Analyses for Cancer and Heart Disease, by Sex and Race, 1969–2014

Variable	ASDR 1969/2014	% Change, 1969−2014	Trend 1		Trend 2		Trend 3		Trend 4		Trend 5	
			Start Year	APC (*P *Value)	Start Year	APC (*P* Value)	Start Year	APC (*P *Value)	Start Year	APC (*P* Value)	Start Year	APC (*P* Value)
**Heart disease**												
All	520.4/166.6	−68.0	1969	–2.8 (<.001)	1977	–1.1 (.02)	1983	–2.4 (<.001)	2002	–4.2 (<.001)	2009	–1.7 (.001)
All men	668.2/211.1	–68.4	1969	–2.3 (<.001)	1978	–1.3 (.04)	1983	–2.7 (<.001)	2002	–4.1 (<.001)	2009	–1.5 (.003)
All women	404.4/131.1	–67.6	1969	–3.1 (<.001)	1977	–0.6 (.21)	1983	–2.2 (<.001)	2002	–4.4 (<.001)	2010	–1.6 (.05)
All white	518.8/165.3	–68.1	1969	–2.8 (<.001)	1977	–1.3 (.01)	1983	–2.5 (<.001)	2002	–4.2 (<.001)	2009	–1.6 (.004)
White men	673.1/210.0	–68.8	1969	–2.0 (<.001)	1987	–4.2 (.03)	1990	–2.1 (<.001)	1997	–3.6 (<.001)	2010	–1.1 (.09)
White women	398.5/129.2	–67.6	1969	–3.0 (<.001)	1977	–0.8 (.15)	1983	–2.3 (<.001)	2002	–4.4 (<.001)	2009	–2.0 (.002)
All black	544.0/207.1	–61.9	1969	–2.5 (<.001)	1977	–0.3 (.27)	1986	–2.0 (<.001)	2002	–4.2 (<.001)	2011	–1.0 (.36)
Black men	643.7/261.6	–59.4	1969	–2.2 (<.001)	1976	–0.4 (.02)	1987	–2.2 (<.001)	2003	–4.1 (<.001)	2011	–0.7 (.52)
Black women	463.7/167.9	–63.8	1969	–3.2 (<.001)	1976	–0.3 (.15)	1986	–1.8 (<.001)	2002	–4.6 (<.001)	2011	–1.2 (.33)
**Cancer**												
All	198.6/161.2	–18.8	1969	0.2 (.32)	1974	0.5 (<.001)	1990	–0.3 (.57)	1993	–1.1 (<.001)	2002	–1.5 (<.001)
All men	247.6/193.3	–21.9	1969	0.8 (<.001)	1980	0.3 (<.001)	1990	–0.5 (.38)	1993	–1.5 (<.001)	2001	–1.8 (<.001)
All women	163.2/137.8	–15.6	1969	–0.3 (.03)	1975	0.6 (<.001)	1990	–0.2 (.55)	1994	–0.8 (<.001)	2002	–1.4 <.001
All white	196.2/161.7	–17.6	1969	0.1 (.60)	1974	0.4 (<.001)	1991	–0.9 (<.001)	2001	–1.4 (<.001)	—	—
White men	244.5/193.3	–20.9	1969	0.7 (<.001)	1980	0.2 (<.001)	1992	–1.4 (<.001)	2002	–1.7 (<.001)	—	—
White women	161.9/138.4	–14.5	1969	–0.3 (.076)	1975	0.6 (<.001)	1991	–0.6 (<.001)	2001	–1.3 (<.001)	—	—
All black	226.0/186.3	–17.6	1969	1.2 (<.001)	1984	0.7 (<.001)	1991	–0.8 (.04)	1995	–1.6 (<.001)	2001	–2.1 (<.001)
Black men	289.7/233.8	–19.3	1969	1.9 (<.001)	1982	1.0 (<.001)	1990	–0.2 (.79)	1993	–2.0 (<.001)	2001	–2.7 (<.001)
Black women	176.3/156.9	–11.0	1969	0.1 (.82)	1975	1.0 (<.001)	1991	–0.6 (.004)	1999	–1.6 (<.001)	—	—

Abbreviations: —, no change from trend 4; APC, annual percentage change; ASDR, age-standardized death rates, expressed per 100,000 persons; PC, percentage change.

From 1969 through 2014, the overall cancer death rates declined among men (21.9%) and women (15.6%). By race and sex, the percentage change declined by 20.9% among white men, 14.5% among white women, 19.3% among black men, and 11.0% among black women. The APCs for cancer death rates increased between 1969 and 1990–1992 in men and women of both racial groups before declining in all groups beginning in the early 1990s through 2014.

In 1969, the risk of heart disease death (measured by the ASDR) was 2.8 times higher than the risk of cancer death among white men and 2.2 times higher among black males. In 2014, the risk of heart disease death was 1.1 times higher than the risk of cancer death among black men and lower (0.9) among white women. In 2009, the cancer death rate surpassed that for heart disease among white women while actual deaths remained higher.

**Figure 1:**
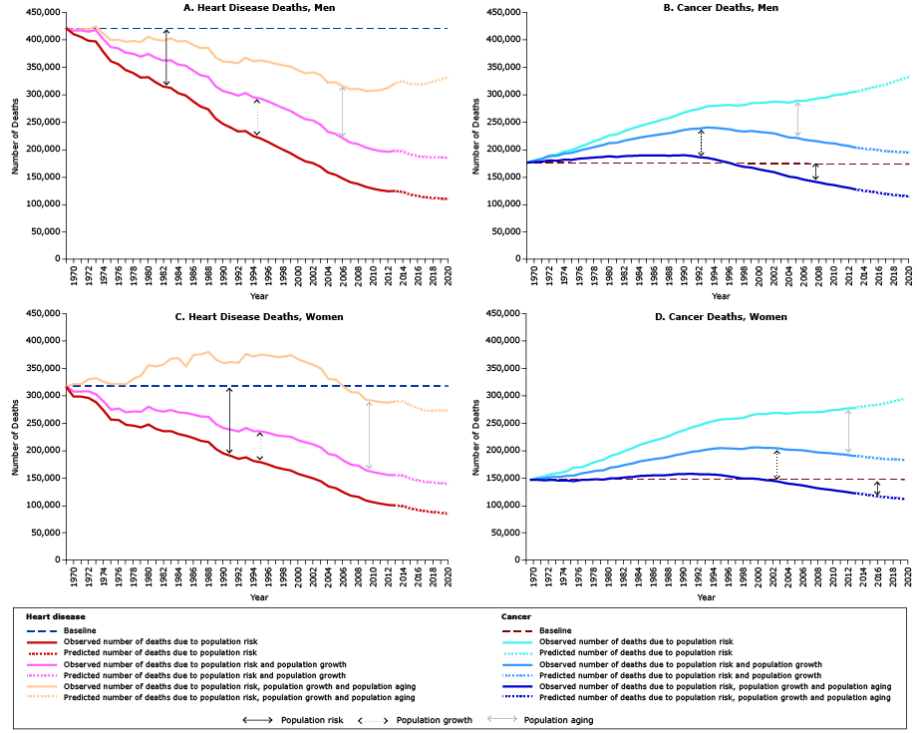
Trends in observed (1969–2014) and predicted (2015–2020) heart disease and cancer deaths attributed to the average person’s risk of dying from the disease (ie, population risk, accounting for such factors as changes in diagnostic and treatment practices), population growth, and population aging, by sex. The blue dashed line (baseline) is the number of deaths from heart disease or cancer that occurred in 1969. The dark yellow (1969–2014) and light yellow (2015–2020) line represents the total number of deaths that would have occurred each year if the population size and age structure remained the same as it was in 1969; this line reflects the effect of changes in population risk. The black (1969–2014) and gray (2015–2020) line represents the total number of deaths that would have occurred if the age structure had remained the same as it was in 1969; this line reflects the effect of changes in risk and population growth. The dark orange (1969–2014) and light orange (2015–2020) line represents the expected number of deaths that actually occurred and thus reflects the combined impact of changes in population risk, growth, and aging. A. Heart disease deaths among men. The number of heart disease deaths attributed to risk declined while the number of heart disease deaths resulting from population growth and aging increased. Observed heart disease deaths declined from 1969 through 2014 and are predicted to increase through 2020, primarily because of an aging population. B. Number of cancer deaths among men. The number of cancer deaths attributed to risk increased from 1969 through 2000 and declined from 2000 forward. The number of cancer deaths resulting from population growth and aging increased. Observed cancer deaths increased from 1969 through 2014 and are predicted to continue to increase through 2020, primarily because of an aging population. C. Number of heart disease deaths among women. The number of heart disease deaths attributed to risk declined while the number of heart disease deaths resulting from population growth and aging increased. Observed heart disease deaths increased from 1969 through 1995, primarily because of an aging population. Observed heart disease deaths are predicted to continue to decrease through 2020, primarily because of continued risk reduction. D. Number of cancer deaths among women. The number of cancer deaths attributed to risk increased from 1969 through 2000 and declined from 2000 forward. The number of cancer deaths resulting from population growth and aging increased. Observed cancer deaths increased from 1969 through 2015 and are predicted to continue to increase through 2020, primarily because of an aging population.

Table 2 and Figure 1 show the contributions to the changes in the total observed (1969–2014) and predicted (2015–2020) number of heart disease and cancer deaths by sex and year attributed to changes in population risk, growth, and aging by sex from the baseline (1969) through 2020. Compared with 1969, the number of heart disease deaths in 2020 is predicted to decrease by 21.3% among men (–73.9% risk, 17.9% growth, 34.7% aging) and 13.4% among women (–73.3% risk, 17.1% growth, 42.8% aging). Cancer deaths are predicted to increase by 91.1% among men (–33.5% risk, 45.6% growth, 79.0% aging) and 101.1% among women (–23.8% risk, 48.8% growth, 76.0% aging). In 2017, more cancer deaths are predicted than heart disease deaths in men (321,107 vs 319,793). In 2015, similar numbers of cancers deaths (281,683) as heart disease deaths (281,675) are predicted for women.

**Table 2:** Observed (1969–2014) and Predicted (2015–2020) Deaths From Heart Disease and Cancer, by Sex, Apportioned Into Changes Resulting From Population Risk, Population Growth, or Population Aging Relative to 1969 (Baseline)^a^

Year of Death	Male				Female			
	Total	Risk^b^	Growth^c^	Aging^d^	Total	Risk^b^	Growth^c^	Aging^d^
**Heart disease**								
1969	421,729	0	0	0	317,341	0	0	0
1975	399,436	−60,516	25,660	12,562	323,216	−61,349	18,193	49,030
1980	405,574	−89,778	42,441	31,182	355,364	−70,163	32,410	75,776
1985	398,101	−123,117	54,444	45,045	353,282	−90,515	41,505	84,951
1990	360,729	−175,024	60,056	53,968	359,225	−123,027	46,308	118,603
1995	362,663	−201,350	72,780	69,504	374,807	−139,128	56,541	140,053
2000	344,766	−236,214	76,871	82,379	365,935	−160,387	61,432	147,549
2005	322,816	−267,501	74,560	94,027	329,238	−186,281	59,671	138,507
2010	307,365	−292,876	71,356	107,156	290,296	−212,098	54,998	130,055
2013	321,329	−297,415	73,479	123,535	289,753	−217,777	55,393	134,796
2014	325,050	−298,886	74,109	128,098	289,255	−219,159	55,731	135,342
2015^e^	319,035	−303,795	72,845	128,257	281,675	−223,641	54,188	133,787
2016^e^	319,315	−306,212	72,940	130,858	278,222	−226,132	53,878	133,135
2017^e^	319,793	−308,629	72,967	133,727	274,913	−228,624	53,511	132,685
2018^e^	324,027	−309,659	73,837	138,121	274,964	−229,917	53,823	133,717
2020^e^	331,711	−311,719	75,471	146,230	274,897	−232,504	54,354	135,706
Change from 1969 to 2020, %	−21.3	−73.9	17.9	34.7	−13.4	−73.3	17.1	42.8
**Cancer**								
1969	175,404	0	0	0	146,360	0	0	0
1975	198,586	5,438	12,849	4,895	171,143	−2,128	10,245	16,666
1980	225,943	12,260	24,002	14,276	190,554	2,939	19,570	21,685
1985	246,917	14,237	34,561	22,715	214,646	7,913	28,238	32,135
1990	268,292	14,723	46,283	31,882	237,047	10,734	37,450	42,504
1995	281,635	4,331	59,391	42,509	256,852	8,839	49,257	52,396
2000	286,072	−10,514	68,316	52,866	267,008	1,894	58,041	60,713
2005	290,417	−25,278	72,560	67,731	268,886	−8,046	62,978	67,594
2010	301,032	−38,505	75,803	88,331	273,706	−18,104	67,000	78,450
2013	307,553	−46,564	76,036	102,676	277,319	−23,957	67,769	87,146
2014	311,285	−48,486	76,568	107,799	280,401	−25,333	68,699	90,675
2015^e^	315,189	−50,251	77,304	112,733	281,683	−27,350	68,825	93,848
2016^e^	317,789	−52,385	77,677	117,093	283,162	−29,144	69,241	96,706
2017^e^	321,107	−54,519	77,990	122,232	285,294	−30,939	69,618	100,255
2018^e^	326,119	−55,911	78,728	127,898	288,432	−32,231	70,265	104,038
2020^e^	335,283	−58,695	80,066	138,508	294,297	−34,814	71,466	111,285
Change from 1969 to 2020, %	91.1	−33.5	45.6	79.0	101.1%	−23.8	48.8	76.0

a Values are number unless otherwise noted.

b Changes in deaths because of change in population risk of death.

c Changes in deaths because of change in population growth.

d Changes in deaths because of change in population aging.

e Predicted values.

**Table:** 

Figure 1A. Men, Heart Disease Deaths				
Year	Baseline	Line 1, Population Size	Line 2, Age Structure	Line 3, Observed Deaths
1969	421,729	421,729	421,729	421,729
1970	421,729	417,803	416,660	410,665
1971	421,729	419,612	417,322	405,475
1972	421,729	418,977	415,111	398,428
1973	421,729	424,390	417,833	397,265
1974	421,729	411,434	401,383	378,257
1975	421,729	399,436	386,874	361,213
1976	421,729	400,533	384,595	355,684
1977	421,729	396,406	376,718	345,037
1978	421,729	398,130	374,602	339,654
1979	421,729	396,281	369,428	331,359
1980	421,729	405,574	374,392	331,951
1981	421,729	400,456	367,489	322,598
1982	421,729	398,469	362,366	315,049
1983	421,729	402,656	362,827	312,503
1984	421,729	397,029	355,000	303,036
1985	421,729	398,101	353,056	298,612
1986	421,729	390,793	343,889	288,042
1987	421,729	385,151	335,640	278,512
1988	421,729	385,337	332,782	273,531
1989	421,729	368,145	315,412	256,697
1990	421,729	360,729	306,761	246,705
1991	421,729	359,740	303,011	240,318
1992	421,729	357,492	298,382	233,184
1993	421,729	367,433	303,233	233,771
1994	421,729	361,229	295,448	224,891
1995	421,729	362,663	293,159	220,379
1996	421,729	360,021	287,981	213,860
1997	421,729	356,549	281,854	206,700
1998	421,729	353,860	276,024	199,940
1999	421,729	351,580	270,488	193,540
2000	421,729	344,766	262,387	185,515
2001	421,729	339,048	255,260	178,601
2002	421,729	340,899	253,166	175,466
2003	421,729	336,068	245,684	168,858
2004	421,729	321,941	232,427	158,177
2005	421,729	322,816	228,789	154,228
2006	421,729	315,676	220,726	147,327
2007	421,729	309,800	212,746	140,634
2008	421,729	311,183	210,046	137,529
2009	421,729	307,202	204,114	132,481
2010	421,729	307,365	200,209	128,853
2011	421,729	308,376	197,455	126,075
2012	421,729	312,462	196,027	124,171
2013	421,729	321,329	197,794	124,314
2014	421,729	325,050	196,952	122,843
2015	421,729	319,035	190,779	117,934
2016	421,729	319,315	188,457	115,517
2017	421,729	319,793	186,066	113,100
2018	421,729	324,027	185,907	112,070
2019	421,729	327,632	185,713	111,040
2020	421,729	331,711	185,481	110,010

**Table:** 

Figure 1B. Men, Cancer Deaths				
Year	Baseline	Line 1, Population Size	Line 2, Age Structure	Line 3, Observed Deaths
1969	175,404	175,404	175,404	175,404
1970	175,404	179,352	179,026	176,437
1971	175,404	183,072	182,404	177,237
1972	175,404	188,668	187,418	179,886
1973	175,404	190,467	188,235	178,978
1974	175,404	195,852	192,267	181,188
1975	175,404	198,586	193,691	180,842
1976	175,404	204,515	198,004	183,119
1977	175,404	209,500	201,035	184,137
1978	175,404	214,980	204,556	185,466
1979	175,404	220,014	207,663	186,268
1980	175,404	225,943	211,667	187,664
1981	175,404	227,874	212,381	186,431
1982	175,404	233,849	216,339	188,094
1983	175,404	238,367	219,052	188,681
1984	175,404	242,790	221,810	189,332
1985	175,404	246,917	224,202	189,641
1986	175,404	250,558	226,183	189,454
1987	175,404	254,655	228,459	189,569
1988	175,404	258,092	230,224	189,242
1989	175,404	263,323	233,316	189,887
1990	175,404	268,292	236,410	190,127
1991	175,404	272,396	238,370	189,046
1992	175,404	274,843	238,839	186,657
1993	175,404	279,401	240,948	185,750
1994	175,404	280,479	240,397	182,980
1995	175,404	281,635	239,126	179,735
1996	175,404	281,916	237,773	176,578
1997	175,404	281,128	235,037	172,371
1998	175,404	282,079	233,839	169,392
1999	175,404	285,826	234,939	168,100
2000	175,404	286,072	233,206	164,890
2001	175,404	287,068	232,112	162,393
2002	175,404	288,763	230,698	159,898
2003	175,404	287,982	226,739	155,826
2004	175,404	286,824	223,253	151,948
2005	175,404	290,417	222,686	150,126
2006	175,404	290,064	219,469	146,488
2007	175,404	292,853	217,699	143,919
2008	175,404	295,253	216,094	141,504
2009	175,404	296,758	213,589	138,636
2010	175,404	301,032	212,696	136,899
2011	175,404	302,228	209,973	134,090
2012	175,404	305,661	208,108	131,823
2013	175,404	307,553	204,901	128,782
2014	175,404	311,285	203,486	126,918
2015	175,404	315,189	202,456	125,153
2016	175,404	317,789	200,696	123,019
2017	175,404	321,107	198,875	120,885
2018	175,404	326,119	198,221	119,493
2019	175,404	330,633	197,522	118,101
2020	175,404	335,283	196,775	116,709

**Table:** 

Figure 1C. Women, Heart Disease Deaths				
Year	Baseline	Line 1, Population Size	Line 2, Age Structure	Line 3, Observed Deaths
1969	317,341	317,341	317,341	317,341
1970	317,341	323,583	308,733	300,580
1971	317,341	323,583	308,733	300,580
1972	317,341	330,811	308,430	296,901
1973	317,341	332,565	302,808	288,513
1974	317,341	326,624	289,465	273,093
1975	317,341	323,216	274,186	255,992
1976	317,341	323,216	276,571	255,686
1977	317,341	322,301	269,603	246,669
1978	317,341	331,161	271,022	245,266
1979	317,341	336,811	270,481	242,012
1980	317,341	355,364	279,588	247,178
1981	317,341	353,282	273,173	239,187
1982	317,341	356,947	270,892	234,969
1983	317,341	367,649	273,698	235,300
1984	317,341	367,973	269,445	229,718
1985	317,341	353,282	268,331	226,826
1986	317,341	374,370	265,415	222,399
1987	317,341	375,095	261,799	217,520
1988	317,341	379,711	261,273	215,187
1989	317,341	365,630	247,971	202,403
1990	317,341	359,225	240,622	194,314
1991	317,341	361,015	238,081	189,798
1992	317,341	360,126	234,585	184,587
1993	317,341	375,947	240,916	187,168
1994	317,341	371,109	235,241	180,608
1995	317,341	374,807	234,754	178,213
1996	317,341	373,259	231,805	174,024
1997	317,341	370,357	227,813	169,080
1998	317,341	370,943	226,014	165,883
1999	317,341	373,565	224,764	163,218
2000	317,341	365,935	218,386	156,954
2001	317,341	361,028	214,708	152,879
2002	317,341	356,001	210,856	148,776
2003	317,341	348,986	205,228	143,545
2004	317,341	330,509	193,698	134,298
2005	317,341	329,238	190,731	131,060
2006	317,341	315,920	181,520	123,574
2007	317,341	306,240	174,065	117,383
2008	317,341	305,616	172,098	114,986
2009	317,341	292,183	163,719	108,422
2010	317,341	290,296	160,248	105,243
2011	317,341	288,174	157,598	102,653
2012	317,341	287,211	155,215	100,463
2013	317,341	289,753	154,957	99,564
2014	317,341	289,255	153,913	98,182
2015	317,341	281,675	147,888	93,700
2016	317,341	278,222	145,087	91,209
2017	317,341	274,913	142,229	88,717
2018	317,341	274,964	141,247	87,424
2019	317,341	274,505	140,235	86,130
2020	317,341	274,897	139,191	84,837

**Table:** 

Figure 1D. Women, Cancer Deaths				
Year	Baseline	Line 1, Population Size	Line 2, Age Structure	Line 3, Observed Deaths
1969	146,360	146,360	146,360	146,360
1970	146,360	150,023	148,394	146,419
1971	146,360	152,896	149,634	145,698
1972	146,360	157,293	152,207	146,521
1973	146,360	159,096	152,178	145,001
1974	146,360	163,073	154,030	145,331
1975	146,360	171,143	154,477	144,232
1976	146,360	171,143	158,342	146,392
1977	146,360	175,372	160,429	146,771
1978	146,360	180,119	163,035	147,530
1979	146,360	183,374	164,138	146,863
1980	146,360	190,554	168,869	149,299
1981	146,360	194,206	170,643	149,415
1982	146,360	199,920	174,104	151,021
1983	146,360	204,590	176,543	151,768
1984	146,360	210,698	180,324	153,729
1985	146,360	214,646	182,511	154,273
1986	146,360	218,807	184,705	154,775
1987	146,360	222,277	186,159	154,665
1988	146,360	226,964	188,737	155,437
1989	146,360	232,857	192,192	156,887
1990	146,360	237,047	194,543	157,094
1991	146,360	242,288	197,603	157,540
1992	146,360	245,743	199,130	156,696
1993	146,360	250,523	201,577	156,612
1994	146,360	253,858	203,425	156,188
1995	146,360	256,852	204,456	155,199
1996	146,360	257,652	204,019	153,155
1997	146,360	258,476	203,380	150,951
1998	146,360	259,490	202,886	148,922
1999	146,360	264,003	205,151	148,973
2000	146,360	267,008	206,295	148,254
2001	146,360	266,692	205,422	146,253
2002	146,360	268,501	205,425	144,952
2003	146,360	268,908	204,225	142,840
2004	146,360	267,056	201,794	139,900
2005	146,360	268,886	201,292	138,314
2006	146,360	269,816	200,756	136,659
2007	146,360	270,014	199,067	134,240
2008	146,360	270,207	196,980	131,595
2009	146,360	270,856	195,772	129,647
2010	146,360	273,706	195,263	128,256
2011	146,360	274,457	193,559	126,200
2012	146,360	276,946	192,620	124,673
2013	146,360	277,319	190,321	122,286
2014	146,360	280,401	189,726	121,027
2015	146,360	281,683	187,835	119,010
2016	146,360	283,162	186,456	117,216
2017	146,360	285,294	185,039	115,421
2018	146,360	288,432	184,394	114,129
2019	146,360	291,280	183,719	112,838
2020	146,360	294,297	183,012	111,546

**Figure 2:**
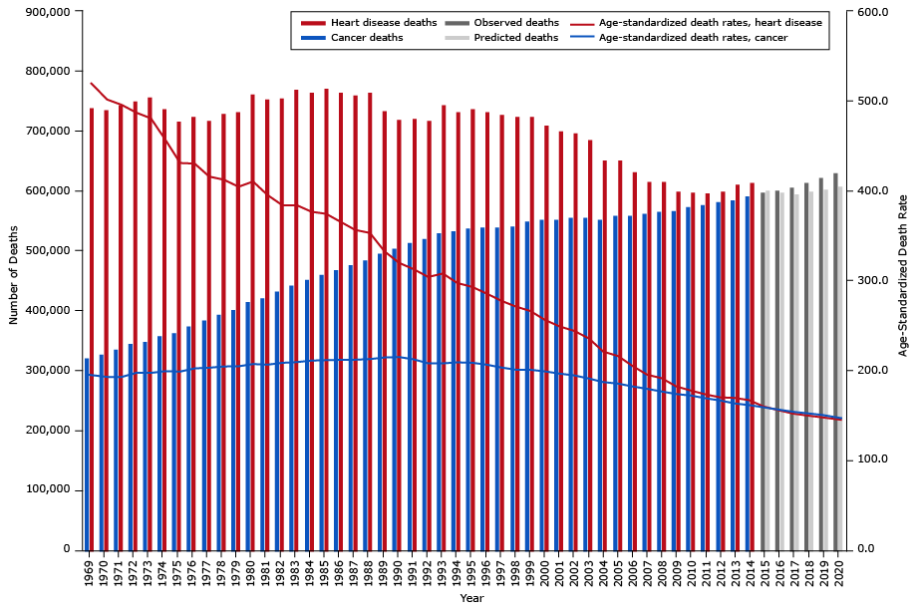
Age-standardized death rates (ASDR) and the observed and predicted number of cancer and heart disease deaths from 1969 through 2020 for men and women combined. **Table:** 

Figure 2 shows the observed (1969–2014) and predicted (2015–2020) number of deaths and death rates for heart disease and cancer by year for all races and both sexes combined. From 2015 to 2020, we predict the number of heart disease deaths overall to stabilize and cancer deaths to increase and surpass heart disease deaths. For 2016, we predict more deaths from cancer than from heart disease (591,426 vs 587,329). In 2020, we predict a total of 627,620 cancer deaths vs 572,415 heart disease deaths.

Year	Cancer		Heart Disease	
	Age-Standardized Death Rate	No. of Deaths	Age-Standardized Death Rate for	No. of Deaths
**Observed**				
1969	198.6	321,764	520.4	739,070
1970	198.8	329,375	502.6	735,363
1971	198.9	335,968	496.6	743,195
1972	200.4	345,961	488	749,788
1973	198.7	349,563	481.7	756,955
1974	199.9	358,925	457.4	738,058
1975	199.1	364,095	431.7	716,098
1976	202.3	375,658	431	723,749
1977	203	384,872	416.6	718,707
1978	204.4	395,099	413	729,291
1979	204.5	403,388	405.2	733,092
1980	207	416,497	411.1	760,938
1981	206.4	422,080	398.8	753,738
1982	208.3	433,769	390.7	755,416
1983	209.2	442,957	390.6	770,305
1984	210.9	453,488	380.4	765,002
1985	211.3	461,563	376.5	770,891
1986	211.8	469,365	366.7	765,163
1987	211.9	476,932	357.4	760,246
1988	212.6	485,056	353.7	765,048
1989	214.3	496,180	333.0	733,775
1990	214.9	505,339	320.3	719,954
1991	215.1	514,684	313.0	720,755
1992	213.5	520,586	304.4	717,618
1993	213.4	529,924	308.3	743,380
1994	211.7	534,337	297.5	732,338
1995	209.9	538,487	293.3	737,470
1996	207.0	539,568	285.9	733,280
1997	203.6	539,604	277.7	726,906
1998	200.8	541,569	271.3	724,803
1999	200.7	549,829	266.4	725,145
2000	198.8	553,080	256.3	710,701
2001	196.3	553,760	249.0	700,076
2002	194.4	557,264	244.1	696,900
2003	190.9	556,890	235.7	685,054
2004	186.8	553,880	220.9	652,450
2005	185.2	559,303	216.1	652,054
2006	182.0	559,880	204.8	631,596
2007	179.3	562,867	195.2	616,040
2008	176.3	565,460	191.3	616,799
2009	173.4	567,614	181.9	599,385
2010	171.7	574,738	177.2	597,661
2011	168.7	576,685	173.1	596,550
2012	166.3	582,607	169.8	599,673
2013	163.0	584,872	169.1	611,082
2014	161.2	591,686	166.6	614,305
**Predicted**				
2015	158.7	596,872	159.4	600,711
2016	156.2	600,951	155.5	597,537
2017	153.7	606,401	151.5	594,707
2018	151.9	614,551	149.5	598,991
2019	150.2	621,914	147.4	602,137
2020	148.4	629,581	145.3	606,608

## Discussion

Our projections indicate that cancer will soon become the leading cause of death in the United States if trends in risk of death from cancer and heart disease and population growth and aging continue. In 1969, there were more than twice as many heart disease deaths as cancer deaths. The decline in heart disease rates (risk) began earlier and was steeper than the decline in risk of death from cancer, which occurred approximately 20 years later. The magnitude of heart disease risk reduction has offset the increase in heart disease deaths from population growth and aging, while the decline in risk of cancer deaths only partially offset the increase in cancer deaths resulting from demographic changes related to population growth and aging. These findings are similar among black and white Americans.

Several factors contributed to the decline in heart disease risk. In 1964, the first Surgeon General’s report on smoking and health (13) linked smoking and lung cancer, and a later report linked smoking with the risk of heart attack and stroke, noting that smokers had about twice the risk of dying from heart disease than lifetime nonsmokers (14). Among smokers, the reduction in excess risk of death from heart disease occurs soon after cessation and is reduced by about half after only one year of smoking abstinence (15). After 15 years of cessation, the risk of death is slightly elevated, but similar to those who never smoked, supporting the hypothesis that the inflammatory component of cardiovascular disease is reversible. Although the declining risk of dying from heart disease has paralleled the decline in smoking prevalence, treatment of cardiovascular disease risk factors has also improved. Cohorts who were aged 50 to 60 years in the 1970s had a 43% lower 10-year cumulative mortality than similar cohorts who reached that age in the 1950s, with significant reductions in cardiovascular disease risk factors such as lower serum cholesterol levels, lower systolic blood pressure, and better overall blood pressure control (16). From 1980 to 2000, approximately half of the decline in heart disease death was attributed to improved treatment after acute myocardial infarction or unstable angina, secondary prevention post-myocardial infarction, treatment of heart failure, and revascularization for chronic angina. The remaining decline was attributed to further reductions in the major risk factors — total cholesterol levels, high blood pressure, and smoking — and increased physical activity (17). Further reduction in the risk of heart disease deaths may have been tempered by increases in body mass index and the prevalence of diabetes (18).

The overall risk of death from heart disease declined in both black and white Americans and, based on our model, is predicted to continue to decline through 2020. Since 1969, the reduction in risk among men has more than offset the increase in heart disease deaths caused by population growth and aging. This reduction in risk has resulted in an overall decline in the observed number of heart disease deaths. However, we predict that the number of heart disease deaths will stabilize or increase slightly in men from 2015 to 2020 as further risk reduction is no longer able to offset the increase in heart disease deaths caused by demographic trends, particularly trends in population aging. A reduction in the number of heart disease deaths among women began more recently and is predicted to continue through 2020.

The overall risk of dying from cancer increased throughout much of the latter part of the last century and is consistent with an increase in the incidence of 4 leading cancers: cancers of the lung and bronchus, colon and rectum, prostate, and female breast (8). Collectively, these cancers accounted for almost 50% of all cancer deaths in the United States. The cancer death rate began to decline in the early 1990s and was largely driven by a decline in deaths from cancers of the lung and prostate in men, breast cancer in women, and a continued decline in colorectal cancer deaths in both men and women that began in the mid-1980s. Lung cancer death rates in women began to decline in the early 2000s, approximately a decade after the decline began in men.

The decline in lung cancer death rates in both men and women parallels a reduction in tobacco use in each group, offset by a latency period of several decades (19). Death rates from other tobacco-related cancers have declined as well, likely the result of reduced smoking prevalence as subsequent Surgeon General reports found convincing evidence for a direct causal relationship (20). Access to quality health care, including early diagnosis through screening, timely follow-up, and evidence-based treatments, is believed to have resulted in increased survival accompanied by reduced mortality from colorectal cancer and, to a lesser extent, female breast cancer and prostate cancer (21).

The risk of dying from cancer increased among men and women throughout the early 1990s before declining, with further risk reduction predicted through 2020. The increase in the risk of death from cancer from 1969 to the early 1990s exacerbated the increase in cancer deaths caused by demographic changes in population growth and aging, resulting in an overall increase in the observed number of cancer deaths. As the risk of cancer deaths began to decline, the number of deaths continued to increase, although at a slower rate. Underlying these trends in deaths is the predicted number of incident cases of cancer, which is expected to increase by more than 20% from 2010 through 2020, whereas the overall risk of being diagnosed with cancer is predicted to remain stable (22).

The risk of dying from heart disease and cancer generally increases with age and, as a result, the numbers of heart disease and cancer deaths increase with the growth and aging of the US population. These demographic influences are likely to continue, because the US population is expected to increase by 10% from 2010 to 2020, with the proportion of the population 65 years of age or older increasing from 13% to 16% (5). The reduction in risk of death from cancer started later than the reduction in risk of death from heart disease and has not been as rapid. Our models estimated that the number of deaths from cancer would surpass the number of deaths from heart disease around 2016, because heart disease deaths were predicted to stabilize or increase slightly in men, and cancer deaths were predicted to continue to increase. Heart disease deaths actually increased in 2013 and again in 2014, somewhat earlier than predicted.

These results are subject to several limitations. This study used methods based on age–period–cohort models that identify trends in younger birth cohorts and extrapolate these trends to future older cohorts (12). Studies have validated these methods by using long-term cancer incidence data but not heart disease or cancer death data. These predictions are based on trends in risk during the past 10 to 30 years; trends in recent years may differ from long-term trends. As such, predictions of heart disease and cancer deaths may be overestimates, because the risk components in these models do not account for potential recent advances in primary prevention and treatment or national initiatives, such as CDC’s Screen for Life: National Colorectal Cancer Action Campaign (http://www.cdc.gov/cancer/colorectal/sfl/) or the Million Hearts project (http://millionhearts.hhs.gov/). At the same time, our predictions may be underestimates because the risk component may not fully account for the potential effect of increased prevalence of obesity and diabetes on risk for cardiovascular disease or for cancers such as pancreatic cancer, which is increasing and now is the fourth leading cause of cancer-related deaths in the United States (23).

The population projections used in these predictions are themselves forecasts of the population size and age composition based on assumptions on future births, deaths, and migration. Furthermore, overall life expectancy in the United States is improving even as disparities by race and socioeconomic factors are increasing (24); these trends are likely to affect population projections. Possible misclassification of underlying cause of death may contribute to imprecision in our estimates.

To counter the anticipated growth in the number of heart disease and cancer deaths, increase the health span of an aging population, and reduce the incidence of heart disease and cancer, a greater emphasis on primary prevention and improved treatment is needed. CDC estimates that each year approximately 91,000 premature deaths from heart disease and 84,000 premature deaths from cancer are potentially preventable (25). Heart disease and cancer share numerous related risk factors, including tobacco use, obesity, and physical inactivity. Further reductions in deaths might yet be achievable if Healthy People 2020 objectives related to risk factors, early diagnosis, and access to health care are met (26).

## Post-Test Information

To obtain credit, you should first read the journal article. After reading the article, you should be able to answer the following, related, multiple-choice questions. To complete the questions (with a minimum 75% passing score) and earn continuing medical education (CME) credit, please go to http://www.medscape.org/journal/pcd. Credit cannot be obtained for tests completed on paper, although you may use the worksheet below to keep a record of your answers. You must be a registered user on Medscape.org. If you are not registered on Medscape.org, please click on the "Register" link on the right hand side of the website to register. Only one answer is correct for each question. Once you successfully answer all post-test questions you will be able to view and/or print your certificate. For questions regarding the content of this activity, contact the accredited provider, CME@medscape.net. For technical assistance, contact CME@webmd.net. American Medical Association’s Physician’s Recognition Award (AMA PRA) credits are accepted in the US as evidence of participation in CME activities. For further information on this award, please refer to http://www.ama-assn.org/ama/pub/about-ama/awards/ama-physicians-recognition-award.page. The AMA has determined that physicians not licensed in the US who participate in this CME activity are eligible for ***AMA PRA Category 1 Credits™***. Through agreements that the AMA has made with agencies in some countries, AMA PRA credit may be acceptable as evidence of participation in CME activities. If you are not licensed in the US, please complete the questions online, print the AMA PRA CME credit certificate and present it to your national medical association for review.

## Post-Test Questions

### Study Title: Heart Disease and Cancer Deaths — Trends and Projections in the United States, 1969–2020

#### CME Questions

1. You are advising a large public health department about expected mortality rates in coming years. According to the review of mortality data, population estimates, and population projections by Weir and colleagues, which of the following statements about the overall effects of risk reduction and population growth and aging on the projected number of heart disease and cancer deaths to the year 2020 is ***correct***?
  - Between 1969 and 2020, the number of heart disease deaths is predicted to decrease by 21.3% among men (−73.9% risk, 17.9% growth, 34.7% aging)
  - Between 1969 and 2020, the number of heart disease deaths is predicted to decrease by 7.4% among women
  - Between 1969 and 2020, the number of cancer deaths is predicted to increase by 27.1% among men
  - Between 1969 and 2020, the number of cancer deaths is predicted to increase by 53.3% among women
2. According to the review of mortality data, population estimates, and population projections by Weir and colleagues, which of the following statements about factors contributing to the decline in heart disease risk is ***correct***?
  - Reducing risk from smoking only occurs after 20 years of cessation
  - The inflammatory component of cardiovascular disease is irreversible
  - Improved treatment of cardiovascular risk factors has had a marked impact
  - From 1980–2000, approximately 20% of decline in heart disease deaths was due to improved treatment of acute myocardial infarction (MI), unstable angina, heart failure, and chronic angina; and secondary prevention after MI
3. According to the review of mortality data, population estimates, and population projections by Weir and colleagues, which of the following statements about factors associated with the increase in cancer risk is ***correct***?
  - Overall risk for cancer deaths increased in parallel with an increase in 4 leading cancers: melanoma, liver, lung, and breast
  - Decline in lung cancer death rates in both sexes parallels a reduction in tobacco use, offset by a latency period of several decades
  - Cancer death rate began to increase in the early 1990s
  - Between 2010 and 2020, the predicted number of incident cases of cancer is expected to remain stable, but the overall risk of being diagnosed with cancer is predicted to increase by more than 20%

**Table:** Evaluation

**1. The activity supported the learning objectives.**				
**Strongly Disagree**				**Strongly Agree**
1	2	3	4	5
**2. The material was organized clearly for learning to occur.**				
**Strongly Disagree**				**Strongly Agree**
1	2	3	4	5
**3. The content learned from this activity will impact my practice.**				
**Strongly Disagree**	** **	** **	** **	**Strongly Agree**
1	2	3	4	5
**4. The activity was presented objectively and free of commercial bias.**				
**Strongly Disagree**				**Strongly Agree**
1	2	3	4	5
